# Editorial: Heme Oxygenases: Novel Regulators of Reproductive Processes

**DOI:** 10.3389/fphar.2015.00282

**Published:** 2015-11-27

**Authors:** Ronald J. Wong, Ana C. Zenclussen

**Affiliations:** ^1^Division of Neonatal and Developmental Medicine, Department of Pediatrics, Stanford University School of MedicineStanford, CA, USA; ^2^Experimental Obstetrics and Gynecology, Medical Faculty, Otto-von-Guericke University, MagdeburgMagdeburg, Germany

**Keywords:** *Hmox1*, HO-1, placenta, uterus, pregnancy, immune system, pre-eclampsia

Heme oxygenase (HO) is a ubiquitous enzyme with various properties, but its main function is catalyzing the rate-limiting step in heme degradation to produce equimolar quantities of biliverdin, iron, and carbon monoxide (CO) (Tenhunen et al., 1968). Of its three isozymes, HO-2 and HO-3 are constitutively-expressed and HO-1 is inducible and acts as stress-response protein. It is not only cytoprotective (Vile et al., 1994; Soares et al., 1998; Gozzelino et al., 2010); but also, exerts anti-inflammatory effects (Otterbein et al., 2000, 2003; Soares and Bach, 2009). Together with its modulatory effects on cell proliferation (Duckers et al., 2001; Lee and Chau, 2002), HO-1 can prevent tissue injury. Also, HO-1 is known to regulate innate and adaptive immunity, and therefore may prevent immune-mediated inflammatory diseases (Wagener et al., 2003; Soares and Bach, 2009; Soares et al., 2009). These effects can be inhibited pharmacologically and restored by CO (Brouard et al., 2000; Otterbein et al., 2000; Lee and Chau, 2002; Ryter et al., 2002; Kim et al., 2006).

Pioneering work from the late Fritz Bach revealed the importance of HO-1 in organ transplantation. Using wild-type (WT, *Hmox1*^+∕+^) and *Hmox1*^−∕−^ mice, Soares et al. (1998) demonstrated that the rapid expression of HO-1 by xenograft endothelial cells, smooth muscle cells, and cardiac myocytes protects xenografts from rejection. The role of HO-1 in xenograft and allograft acceptance is due to its cytoprotective properties that support cell survival and function within the transplanted organ. Moreover, HO-1 can reduce the graft immunogenicity by directly modulating recipient immune response such that regulatory responses are generated. The activation of HO-1 expression in the graft and in immune cells of the recipient can prevent rejection and promote immunotolerance, and probably due to the detoxification of free heme by HO-1 (Soares and Bach, 2007).

Using a mouse model where tolerance is induced by donor-specific transfusion and anti-CD40L, Yamashita et al. (2006) observed that HO-1 is necessary for long-term graft tolerance as grafts do not survive in *Hmox1*^−∕−^ compared to WT control recipients. Modulation of HO-1 was necessary to promote graft tolerance. Donor-specific transfusion alone failed to prolong survival of transplanted hearts, but long-term survival and tolerance were achieved after HO-1 induction. HO-1 induction plus donor-specific transfusion was associated with increases in regulatory T-cells (Tregs) (Yamashita et al., 2006). The immunomodulatory effect on cells from graft recipients is based on the fact that HO-1 directly modulates the phenotype of dendritic cells (DCs) (Moreau et al., 2009). HO-1 is constitutively expressed in immature DCs; however, its expression decreases during DC maturation. HO-1 upregulation can maintain DCs in an immature state, which suppresses the immune response, and then leads to antigen-specific Treg generation (George et al., 2008; Schumacher et al., 2012). Because Tregs from *Hmox1*^−∕−^ mice are functional, it can be concluded that the suppressive function of Tregs depends upon HO-1-induced modulation of DCs rather than HO-1 expression by Tregs (Zelenay et al., 2007).

In the article by Schumacher and Zenclussen (2015), the participation of HO-1 in immunomodulation during pregnancy and organ transplantation is discussed. They report how HO-1 promotes alloantigen tolerance by blocking DC maturation reduces T-cell responses and increases Treg numbers. Further mechanisms involve the cytokine milieu, tissue protection, and apoptosis. Hence, HO-1 can mediate acceptance of a transplanted allogeneic graft through organ cytoprotection and immunotolerance. A similar scenario may be true for a growing fetus, which is semi-allogeneic to the mother, where HO-1 confers both semi-allograft cytoprotection and immunotolerance in the maternal immune system.

Because of its role in the modulation of innate and adaptive immune responses, HO-1 is linked to carcinogenesis by influencing tumor induction, growth, and metastasis (Jozkowicz et al., 2007). HO-1 is highly expressed in several tumors, and accordingly, inhibition of HO may have potential as a therapeutic approach. Because tumors are highly vascularized and prone to massive hemorrhaging, large quantities of free heme can be released, and induce HO-1 that in turn negatively influences the host and protects the tumor from oxidative injury. HO-1 is also involved in tumor angiogenesis and stimulating tumor-associated macrophages (Was et al., 2010), and thus, may regulate tumor survival and progression.

Zhao et al. (2015) discuss how HO-1 regulates similar processes in transplantation and pregnancy, particularly in invasion and neovascularization. Pregnancy is a physiological state characterized by interactions of various processes occurring at different stages. For these changes to occur, tissue and vascular remodeling as well as both pro- and anti-inflammatory processes in the uterus are required. However, once the placenta has formed and the fetus grows, the fetoplacental unit behaves more like a graft that is tolerated by its host. When pregnancy is near completion, the semi-allograft is naturally “rejected” by the mother resulting in birth.

Zenclussen et al. (2015) review how HO-1 impacts reproductive processes, highlighting its importance in placental function and fetal development. The deletion of *Hmox1* in mice leads to inadequate spiral artery remodeling and suboptimal placentation followed by intrauterine growth restriction and fetal death (Zhao et al., 2009; Zenclussen et al., 2011). A partial *Hmox1* deletion is compatible with pregnancy, however heterozygote females develop gestational hypertension (Linzke et al., 2014). The protective effects of HO-1 on placentation and fetal growth can be mimicked by exogenous administration of CO. CO promotes the *in situ* proliferation of uterine natural killer (uNK) cells, restores placentation, and fetal growth, while normalizing blood pressure (Linzke et al., 2014). Similarly, HO-1 inhibition provokes hypertension in pregnant rats (George et al., 2013).

The relevance of HO-1 in the regulation of immune responses during pregnancy is further highlighted in the article by George et al. (2015). They investigated whether HO-1 induction could attenuate TNF-α-induced hypertension in pregnancy. HO-1 induction significantly decreased blood pressure in TNF-α-infused animals, which was accompanied by a normalization of vascular parameters, supporting the notion that HO-1 is essential in counteracting the negative effects of excessive inflammation.

In summary, the HO-1/CO axis may play a pivotal role in sustaining pregnancy, and thus understanding its biology during pregnancy may reveal promising therapeutic approaches for pregnancy complications.

## Author contributions

Both the authors contributed in writing the editorial.

### Conflict of interest statement

The authors declare that the research was conducted in the absence of any commercial or financial relationships that could be construed as a potential conflict of interest.
